# Efficient marmoset genome engineering by autologous embryo transfer and CRISPR/Cas9 technology

**DOI:** 10.1038/s41598-021-99656-4

**Published:** 2021-10-12

**Authors:** Yukiko Abe, Harumi Nakao, Motoki Goto, Moe Tamano, Michinori Koebis, Kazuki Nakao, Atsu Aiba

**Affiliations:** 1 Section of Animal Research and Laboratory of Animal Resources, Center for Disease Biology and Integrative Medicine, Graduate School of Medicine, The University of Tokyo, 7-3-1 Hongo, Bunkyo-ku, Tokyo, 113-0033 Japan; 2 Institute of Experimental Animal Sciences, Graduate School of Medicine, Osaka University, 2-2 Yamadaoka, Suita, Osaka 565-0871 Japan

**Keywords:** Biological techniques, Neuroscience, Neurology

## Abstract

Genetic engineering of non-human primates, which are most closely related to humans, has been expected to generate ideal animal models for human genetic diseases. The common marmoset (*Callithrix jacchus*) is a non-human primate species adequate for the production of genetically modified animals because of their small body size and high reproductive capacity. Autologous embryo transfer (AET) is routinely utilized in assisted reproductive technologies for humans but not for experimental animals. This study has developed a novel method for efficiently producing mutant marmosets using AET and CRISPR/Cas9 systems. The embryos were recovered from oviducts of naturally mated females, injected with Cas9/guide RNA, and transferred into the oviducts of the donors. This AET method can reduce the time for in vitro culture of embryos to less than 30 min. This method uses an embryo donor as the recipient, thus reducing the number of animals and allowing for “Reduction” in the 3R principles of humane experimental technique. Furthermore, this method can utilize nulliparous females as well as parous females. We applied our novel method and generated the 6 marmosets carrying mutations in the fragile X mental retardation 1 (*FMR1*) gene using only 18 females including 14 nulliparous females.

## Introduction

Most genetically engineered animal models for human diseases are generated using mice, which are easy to engineer genetically. On the other hand, genetically engineered mice cannot reproduce all the symptoms of human diseases. Therefore, genetic engineering of non-human primates, which are most closely related to humans, has been expected. In non-human primates, there are several reports of genome editing in the genus *Macaca* such as rhesus and cynomolgus monkeys to produce mutants as animal models of diseases: *MECP2* knockout monkeys using transcription activator-like effector nuclease (TALEN)^1^, *RAG1* and *PPARG* knockout monkeys^2^, *BMAL1* knockout monkeys^3^ and *PKD1* knockout monkeys^4^ using CRISPR/Cas9.

The common marmoset (*Callithrix jacchus*) is a non-human primate species adequate for the production of genetically modified animals because of their small body size and high reproductive capacity^5^. Transgenic and knockout marmosets were successfully generated by virus infection^6,7^ and genome editing methods^8^, respectively.

Conventional methods to generate mutant marmosets include the following steps: (1) collection of oocytes from ovaries from one set of females, called donors, (2) in vitro maturation of oocytes and in vitro fertilization with sperms from males. (3) microinjection of DNA, RNA, or proteins of genome editing methods such as TALENs and CRISPR/Cas9 systems into embryos and culture in vitro for several days, and (4) transfer of injected embryos into the uteri of another set of females, recipient females. The conventional methods require a long period of in vitro culture and many donors and recipients. In addition, since these methods need parous females as recipients, it is difficult to use these methods in laboratories where the number of females is limited.

**Figure 1:**
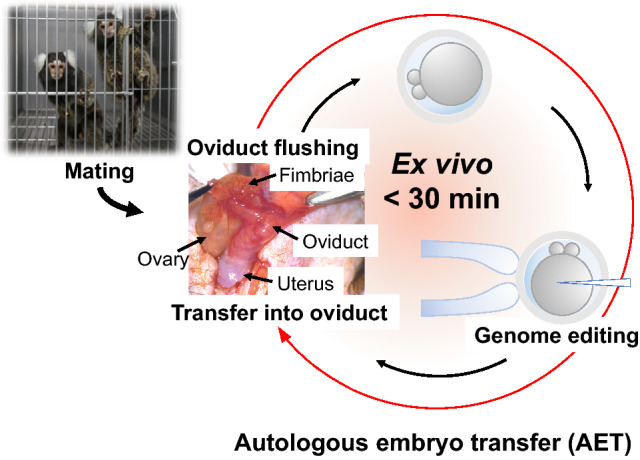
Autologous embryo transfer (AET). Pronuclear stage embryos are recovered from the oviducts of a naturally mating female by flushing. Immediately embryos are injected with Cas9 protein and guide RNAs. Injected embryos are transferred into the oviduct of the female providing the embryos. Injected embryos are exposed ex vivo for less than 30 min.

Autologous embryo transfer (AET) is routinely utilized in human-assisted reproductive technologies to treat infertility. On the other hand, the application of AET to laboratory animals is limited. AET was utilized to generate mutant dog^9^, but not for non-human primates. This study has developed a novel method for efficiently producing mutant marmosets using AET combined with the CRISPR/Cas9 system (Fig. 1). First, we collected pronuclear stage embryos from naturally mated females. Secondly, the genome-editing embryos were transferred autologously into oviducts of the females that provided embryos. Importantly, we can utilize nulliparous females as recipients. This AET method can reduce the time for in vitro culture of embryos to less than 30 min, resulting in high efficiency for the development of injected embryos. Furthermore, this method uses an embryo donor animal as the recipient animal, thus reducing the number of animals and allowing for “Reduction” in the 3R principles of humane experimental technique. As far as we know, this is the first report of the production of newborns by AET in non-human primates.

The fragile X syndrome is recognized as the most common monogenic cause of intellectual disability and autism spectrum disorder and caused by expansion of CGG triplet repeats in the 5′ untranslated region of the fragile X mental retardation 1 (*FMR1*) gene encoding the fragile X mental retardation protein (FMRP)^10^. In the patient with a full mutation (> 200 repeats), transcription of *FMR1* is silenced, and FMRP is decreased^11^, leading to symptoms such as intellectual disability, autism spectrum disorder, seizure, and developmental delays^10^. As a genetic mutant model, *Fmr1* knockout mice were generated^12^. The knockout mice mimicked the symptoms of the fragile X syndrome, including learning deficits, hyperactivity, impaired social behaviors, and susceptibility to audiogenic seizures^12,13^.

We applied the AET method combined with the CRISPR/Cas9 system to generate *FMR1* mutant marmosets. We generated *FMR1* mutants with high efficiency using a small number of females to show the advantage of our novel method.

## Materials and methods

### Animals

Common marmosets (*Callithrix jacchus*) were purchased from CLEA Japan Inc. (Tokyo, Japan) and housed under 12 h light/dark cycle (light on at 08:00, off at 20:00), with free access to food and water, at a temperature maintained at 28 °C. Animals between the ages of 2 and 8 years were used. Estrus cycle was monitored by measurement of the concentration of progesterone ([P4]) in blood using a competitive enzyme immunoassay (ST AIA-PACK PROGII ; Tosoh Co, Tokyo, Japan). The animal experiments were reviewed and permitted by Institutional Animal Care and Use Committee of the University of Tokyo (permission number M-P16-030) and conducted following “Manual for Animal Experiment of the University of Tokyo” and “Guidelines for the Care and Use of Nonhuman Primates in Neuroscience Research” of The Japan Neuroscience Society. The study was also carried out in compliance with the ARRIVE (Animal Research: Reporting of In Vivo Experiments) guidelines for animals.

### Anesthesia

Embryo collection and transfer were carried out under anesthesia. 0.05 mg/kg medetomidine (Domitol; Meiji Seika Pharma, Tokyo, Japan), 0.5 mg/kg midazolam (Dormicum; Astellas Pharma, Tokyo, Japan), and 0.5 mg/kg butorphanol (Vetorphale; Meiji Seika Pharma) were administered before anesthesia was induced by inhaled isoflurane (MSD Animal Health, Madison NJ).

### Validation of guide RNAs

We validated the efficiency of guide RNAs (gRNAs) targeting an exon, which encodes 31 amino acid residues, corresponding to amino acid residues 36–66 of human FMRP protein with 97% identity (FMR1-T5; GAGGTGGGAATCTGACATCATGG). SpCas9/gRNA complex was transfected into the marmoset embryonic stem (ES) cells (CMES40 (AES0166), RIKEN BRC CELL BANK)^14^ using CRISPRMAX transfection reagent (Thermo Fisher Scientific, Waltham, MA). Genomic DNA was extracted from the transfected cells four days after transfection. PCR for *FMR1* locus was performed using primers (5′-GGGGGTCACACTTAACCAAGAGTTGATGGC-3′, 5′-CTAGTGGGCAAAGAAACTTGAGGCAGGGAC-3′) under the following conditions: 94 °C for 2 min, 30 cycles of melting at 98 °C for 10 s, annealing, and extension at 68 °C for 50 s, with additional extension at 68 °C for 2 min at the end. PCR products were digested with resolvase using a Mutation detection kit (Takara Bio USA, Mountain View, CA) and separated in 2% agarose gels.

### Embryo collection, microinjection, and transfer

For embryo collection, we utilized both parous (n = 4) and nulliparous (n = 14) females. To reset the estrus cycle of female marmosets, the prostaglandin analog cloprostenol (Estrumate; MSD Animal Health) was administrated (day 0). On day 1, we confirmed that [P4] was less than 10 ng/ml. From day 6, female marmosets were mated with mature male marmosets. From day 8, vaginas of females were examined daily for sperm to confirm mating. After confirmation of mating, we monitored estradiol concentration ([E2]) and [P4] in blood every day using competitive enzyme immunoassays (ST AIA-PACK iE2 and ST AIA-PACK PROGII; Tosoh Co). Embryos were collected from the oviducts by a slightly modified method previously reported^15^, when [P4] had increased and [E2] had decreased compared to the previous day. Results of hormone concentration are expressed as mean ± SEM.

The oviducts and ovaries were exteriorized by midline laparotomy and placed in a 60-mm culture dish. We inserted a 27-gauge winged needle into isthmus of the oviducts and flushed twice with approximately 2 ml of OptiMEM medium (Thermo Fisher Scientific) containing 9.1 mg/ml hyaluronidase (MilliporeSigma, Munich, Germany), 91 U/ml heparin (Mochida, Tokyo, Japan), and 0.091% polyvinyl alcohol (MilliporeSigma) (Supplementary Video S1). Embryos were cultured in Cleav medium (Origio, Måløv, Denmark) at 38 °C, 5% O_2_, and 5% CO_2_ except during the microinjection.

For injection of marmoset embryos, we prepared SpCas9 protein (100 ng/µl; Takara Bio, Kusatsu, Japan)/crRNA (50 ng/µl; Integrated DNA Technologies (IDT), Tokyo, Japan)/tracrRNA (50 ng/µl; IDT) mixture and SpCas9 protein (100 ng/µl; Takara)/sgRNA (50 ng/µl; Fasmac, Atsugi, Japan) mixture in PBS. The mixture of SpCas9 protein and gRNAs was injected into the cytoplasm of the collected embryos in an M2 medium (MilliporeSigma). Injected embryos were transferred autologously into the oviducts that provided embryos (Supplementary Video S2). Time spent in manipulating embryos in vitro was less than 30 min. One month after embryo transfer, pregnancy was confirmed by ultrasonography. Maintenance of pregnancy was monitored weekly by measuring [P4] in urine.

### Genotyping of mutant marmosets

Genomic DNA was extracted from dozens of hair follicles collected from each newborn or a spermatozoon collected from an adult male. PCR for *FMR1* locus was performed under the same condition as for the validation of the gRNA. The PCR products were analyzed by 2% agarose gel electrophoreses and direct sequencing.

### Isolation of primary fibroblasts from marmosets

Approximately 1 cm of marmoset tails was cut into 1–2 mm pieces with scissors and treated with 300 U/ml collagenases at 37 °C for 90 min. The collagenase-treated tissue was ground with the gasket part of the syringe, and the tissue solution was collected by straining the cells through a 40-μm cell strainer. The cells were then suspended in Dulbecco's Modified Eagle Medium (MilliporeSigma) supplemented with 10% FCS (Cell Culture Technologies, Gravesano, Switzerland), spread on 60-mm dishes, and cultured at 37 °C and 5% CO_2_.

### Western blot analysis

Fibroblasts were homogenized in a buffer containing 20 mM Tris–HCl (pH 7.4), 1 mM EDTA, 1% NP-40, 150 mM NaCl, 0.5% Sodium Deoxycholate, 0.1% SDS and protease inhibitors (cOmplete Mini, EDTA-free; Roche). Whole brains were homogenized in a buffer containing 0.32 M sucrose, 10 mM Tris–HCl (pH 7.4), 1 mM EDTA, and protease inhibitors. Ten micrograms of proteins were separated by SDS-PAGE and transferred to Immobilon-P membrane (Merk Millipore, Burlington, MA). The membrane was probed with rabbit antibodies against the C-terminal sequence of human FMRP (Abcam, ab17722) or a mouse anti-β-actin antibody (MilliporeSigma, A2228) followed by anti-rabbit or anti-mouse HRP-conjugated secondary antibodies. Bound antibodies were visualized with ECL Prime detection reagents (GE Healthcare, Chicago, IL, RPN2232). The blots were imaged using ChemiDoc imaging systems (Bio-Rad, Hercules, CA) or ImageQuant LAS 4000 system (GE Healthcare).

## Results

### Embryo collection

**Figure 2:**
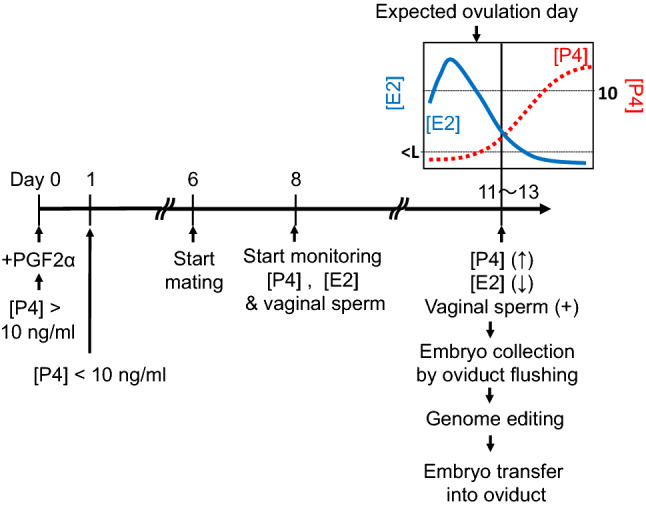
The protocol for genome editing of marmoset using autologous embryo transfer (AET). A schematic diagram of the protocol for collection, genome editing, and transfer of marmoset embryos. The protocol is set to collect a maximal number of pronuclear stage embryos. On day 0, PGF2α analogs are administrated to late luteal phase females with blood [P4] > 10 ng/ml. On day 1, the blood [P4] of PGF2α-treated females is confirmed to be less than 10 ng/ml. On day 6, female marmosets start housed with mature male marmosets. From day 8 onward, blood [P4] and [E2] and vaginal sperm are examined daily. On days 11–13, when [P4] has increased and [E2] has decreased compared to the previous day, embryos are collected from the oviducts of mated females. Immediately after collection, pronuclear stage embryos are injected with Cas9/gRNA complexes and transferred autologously into the oviduct, which provides the embryos.

**Figure 3:**
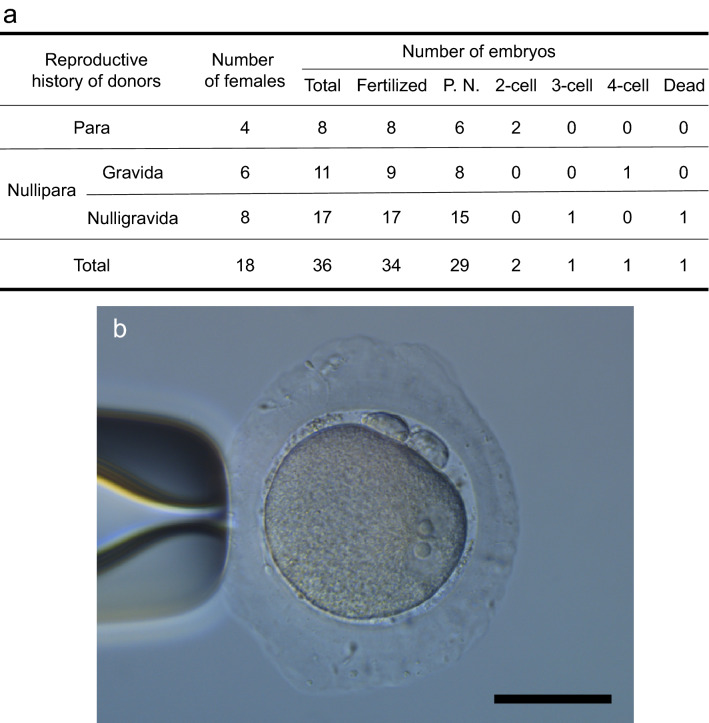
Embryos recovered from the oviduct of naturally mated females. (**a**) Thirty-six embryos were recovered from 18 naturally mated females. Twenty-nine (81%) of the embryos were at the pronuclear stage (P. N.). (**b**) A pronuclear stage embryo collected by flushing an oviduct of a naturally mated female. Two pronuclei are observed. A scale bar, 50 μm.

Because it was reported that marmoset embryos obtained by natural mating were much better developmental potential than embryos obtained by in vitro fertilization^8^, we planned to collect embryos from naturally mated females. We optimized the protocol for the embryo collection to maximize the number of pronuclear stage embryos adequate for mutant generation by genome editing (Fig. 2). Firstly, female marmosets were administered the prostaglandin F2α (PGF2α) analog to reset the estrus cycle (day 0 in Fig. 2). On day 1, progesterone concentration ([P4]) was confirmed to be less than 10 ng/ml. On day 6, female marmosets start mating with mature male marmosets. After confirmation of sperm in vaginas, we monitored estradiol concentration ([E2]) and [P4] in blood every day (Supplementary Table S1). On days 11 -13, when [P4] had increased and [E2] had decreased compared to the previous day, embryos were collected from the oviducts of 18 females ([E2] = 0.21 ± 0.06 ng/ml (n = 7), < detection limit (0.02 ng/ml; n = 11); [P4] = 5.6 ± 1.0 ng/ml (n = 18)). We collected 36 embryos from 18 naturally mated females. Twenty-nine (81%) of the embryos were at the pronuclear stage (Fig. 3a,b). The average number of pronuclear stage embryos per animal was 1.5 and 1.6 from parous (n = 4) and nulliparous (n = 14) females, respectively, suggesting that nulliparous females can be utilized as embryo donors.

### Generation of *FMR1* mutant marmosets

**Figure 4:**
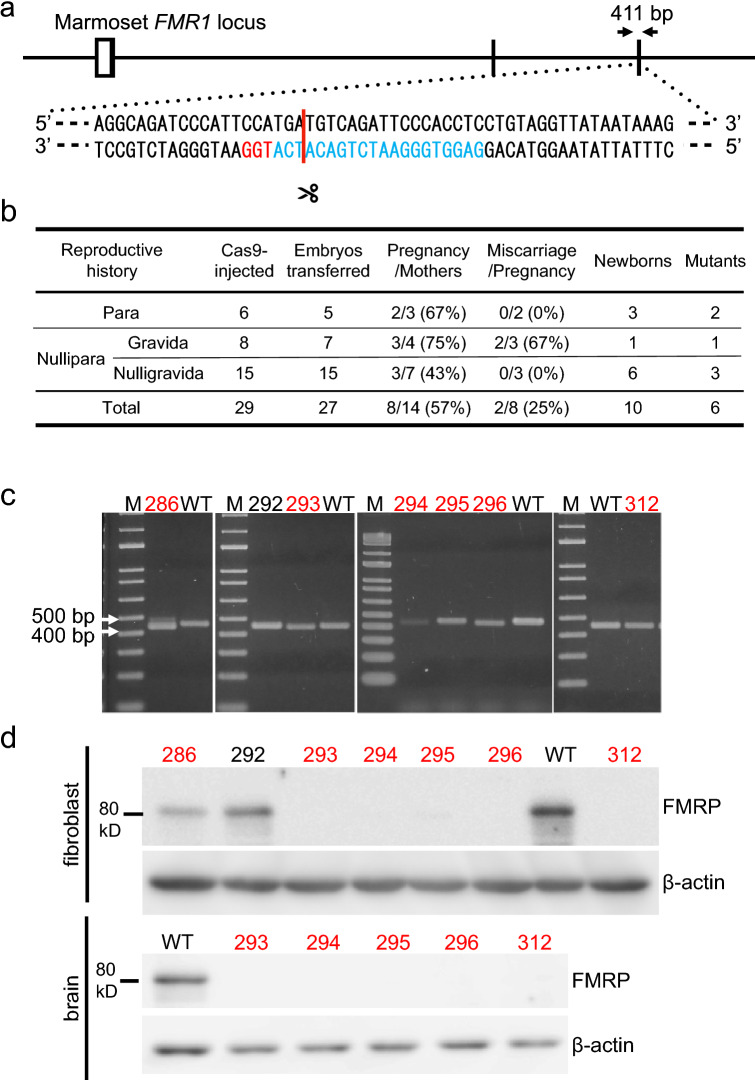
Generation of *FMR1* mutant marmosets. (**a**) A schematic diagram of the *FMR1* locus is shown. A white square indicates a non-coding exon, and black squares indicate coding exons. The positions of double-strand break by Cas9 (red bar) were designed in a coding exon. A protospacer adjacent motif (PAM) and 20 nucleotides adjacent to the PAM are shown in red and blue. Genotyping primers (arrows) produce a 411bp DNA fragment. (**b**) The efficiency of CRISPR/Cas9 to generate mutant marmosets. The injection was performed into the cytoplasm of 29 pronuclear stage embryos, and 6 of the 10 newborns carried *FMR1* mutations. Both parous and nulliparous females efficiently produced mutant marmosets. (**c**) PCR products of newborns (#286, #292, #293, #294, #295, #296, and #312) were separated on 2% agarose gels. Red numbers indicate mutants. As a control, PCR products (411 bp) using genomic DNA extracted from hair follicles of wild-type (WT) marmosets were used. (**d**) Western blot analysis of FMRP and β-actin. Proteins extracted from fibroblasts and whole brains were separated on 8% polyacrylamide gels. All lanes contain 10 μg of protein. The blots cropped from different parts of a same gel were separately shown with a white space.

To establish a non-human primate model of the fragile X syndrome, we planned to introduce null mutations in the *FMR1* gene located on the X chromosome of common marmosets. We designed a gRNA targeted an exon, which encodes 31 amino acid residues, corresponding to amino acid residues 36–66 of human FMRP protein with 97% identity (FMR1-T5; Fig. 4a). To validate the efficiency of the gRNA in the double-strand break at the target site, we transfected the marmoset ES cells with complexes of SpCas9 and crRNA/tracrRNA (FMR1-crT5) or single-guide RNA (FMR1-sgT5). The complexes containing both gRNAs efficiently introduced mutations in the FMR1 locus in the marmoset ES cells (Supplementary Figure S1). Therefore, SpCas9 protein and gRNAs (FMR1-crT5 or FMR1-sgT5) were injected into the cytoplasm of 29 pronuclear stage embryos (Figs. 3b, 4b), and 27 were transferred into the oviduct of the donors. The remaining 2 embryos were cultured in vitro*,* and we confirmed *FMR1* mutations in their genome. One month after embryo transfer, the pregnancy of 8 out of the 14 female marmosets was confirmed by ultrasonography (Fig. 4b). Pregnant rates for parous (2/3 (67%)) and nulliparous females (6/11 (55%)) were similar, suggesting nulliparous females can be utilized as recipients of embryo transfer (Fig. 4b). Maintenance of pregnancy was monitored weekly by measuring [P4] in urine, and we found that only 2 of the 8 pregnancies resulted in miscarriages (Fig. 4b). The remaining 6 females delivered 10 newborns between 143 and 148 days after embryo transfer. We confirmed 6 of 10 newborns to carry *FMR1* mutations by PCR analysis of the genomic DNA and sequencing analysis of the PCR products (Fig. 4b,c, Supplementary Figure S2). We utilized 4 parous, and 14 nulliparous marmosets for this experiment and both could deliver mutant newborns efficiently: 2 and 4 mutants from 4 parous and 14 nulliparous females, respectively. We found that all mutations were small deletion in the *FMR1* coding region (Supplementary Figure S2). Deletion lengths were 15 bp, 7 bp, 21 bp, 1 bp, 20 bp and 1 bp in #286, #293, #294, #295, #296 and #312, respectively (Supplementary Figure S2). The PCR product of #286 male marmoset showed a shifted band in the agarose gel electrophoresis, and at least two waveforms in DNA sequencing analysis, suggesting its mosaicism (Fig. 4c, Supplementary Figure S2). Fifteen bp deletion in #286 and 21 bp deletion #294 are expected to introduce deletion of 5 and 7 amino acid residues of the FMRP, respectively. Seven bp deletion in #293 and 20 bp deletion in #296 are expected to introduce deletion and frameshift mutations, leading to premature termination of translation. The same 1 bp deletion of #295 and #312 is expected to lead to premature termination of translation.

### *FMR1* mutant phenotype

In contrast to no apparent phenotype for neonatal viability of *Fmr1* knockout mice^12^, all the *FMR1* mutant marmosets except #286 male with mosaicism died by 8 days of age. To examine FMRP expression, we carried out western blot analyses of lysates from the brain and fibroblasts of the mutant marmosets using antibodies against the C-terminal sequences of FMRP. The mutants except for #286 showed almost undetectable FMRP protein in the brain or fibroblasts (Fig. 4d, Supplementary Figures S4a, S4b, S5a, S5b). Mosaic animal #286 led to a reduced amount of FMRP proteins in the fibroblasts compared to wild-type fibroblasts. This male marmoset has reached sexual maturity and looks healthy. We decided the DNA sequence of the *FMR1* gene in each spermatozoon collected from #286. We confirmed a germline transmission of the *FMR1* mutant allele containing 15 bp deletion identified in the genome of this mosaic male (Supplementary Figures S2, S3).

## Discussion

This study has developed a novel efficient marmoset genome engineering method using autologous embryo transfer (AET) and CRISPR/Cas9 system. As far as we know, this is the first report of the production of newborns by AET in non-human primates.

The common marmoset is a non-human primate species adequate for mutant animals because of their small body size and high reproductive capacity. In conventional methods of marmoset genome engineering, the injected embryos are transferred to the uterus of parous females. The procedures require donor females in addition to the recipient females. For example, in the first report of the production of mutant marmosets, 250 embryos and 113 recipient females were used to produce 9 *IL2RG* knockout marmosets^8^. To carry out a similar experiment, scientists should probably possess more than 300 animals in their colony. It would be challenging to generate mutant marmosets using the conventional methods in laboratories where the number of females, especially parous females, is limited. This study used 18 females, including 14 nulliparous females, to produce 6 *FMR1* mutants. We think that 100 marmosets in a colony are enough to generate 5 mutant marmosets in a year.

Another advantage of our method is the high developmental potential of genome-engineered embryos. We utilize embryos obtained by natural mating, which are much better developmental potential than embryos obtained by in vitro fertilization^8^. In addition, in vitro culture time in our protocol is much less than conventional methods. It takes less than 30 min to collect pronuclear stage embryos from the oviduct and transfer the genome-edited embryos autologously into the oviduct. On the other hand, using conventional methods takes at least a week or more from oocyte collection to transfer injected embryos to the uterus. We think that the developmental potential of injected embryos using our method is higher than that using the conventional methods because of its shorter in vitro culture time.

Expansion of mutant non-human primates is often hampered by difficulties of germline transmission of mutant alleles. Intracytoplasmic sperm injection (ICSI) of mutant spermatozoa may overcome the problem. Our AET method could also provide the ideal eggs for ICSI if we recovered eggs from the oviduct of unmated females. We would take advantage of the short culture time and high developmental potential of embryos by our method to produce offspring successfully. We plan to obtain *FMR1* mutant females by intracytoplasmic injection of *FMR1* mutant spermatozoa collected from the mosaic male (#286) using our AET method.

Animal experiments using non-human primates should require the strict application of 3R principles of humane experimental technique. Our method uses an embryo-donor animal as the recipient animal, thus reducing the number of animals and allowing for “Reduction” in the 3R principles. We collected embryos from naturally mated females to take advantage of much better developmental potential than embryos obtained by in vitro fertilization, preventing the waste of embryos. Furthermore, we observed few tissue adhesions after laparotomy was performed, probably because a puncture of the oviduct for flushing was only intraperitoneal tissue damage in our method. Therefore, we consider that 3R principles of the humane experimental technique are fully satisfied in our approach.

We have generated the 6 *FMR1* mutant marmosets and found that the 5 mutants expressing undetectable FMRP protein died by 8 days of age in contrast to the overtly normal development of *Fmr1* knockout mice^12^. In most fragile X syndrome patients, abnormal expansions of triplet repeat (CGG) in 5' untranslated region of the *FMR1* gene induce hypermethylation, resulting in suppression of transcription. On the other hand, deletions in the *FMR1* locus were occasionally observed in the patients^16^. Transmission of the *FMR1* alleles containing deletion mutations was traced in some families^16^. Therefore, neonatal death of the *FMR1* knockout observed in this study may be a specific phenotype in the common marmoset. We plan to obtain *FMR1* heterozygous mutant females by crossing #286 and wild-type females or ICSI using AET. Female fragile X syndrome patients carry heterozygous mutations and show milder symptoms than male patients with hemizygous mutations. Thus, female *FMR1* mutant offspring could survive in the neonatal stage to analyze the effects of FMRP decrease in higher brain functions related to symptoms of fragile X syndrome.

## Supplementary Information

Supplementary Video 1.

Supplementary Video 2.

Supplementary Information 1.

Supplementary Information 2.

Supplementary Information 3.

Supplementary Information 4.

Supplementary Information 5.

Supplementary Information 6.

Supplementary Information 7.

## Data Availability

The data that support the findings of this study are available from the corresponding authors, Kazuki Nakao (k_nakao@iexas.med.osaka-u.ac.jp) or Atsu Aiba (aiba@m.u-tokyo.ac.jp), upon reasonable request.
