# Supplementary material for: Efficient marmoset genome engineering by autologous embryo transfer and CRISPR/Cas9 technology
Source: Sci Rep. 2021 Oct 12;11:20234. doi: 10.1038/s41598-021-99656-4 (PMC8511084; doi:10.1038/s41598-021-99656-4)
Supplement: Supplementary file 3 — Supplementary Information 1. [file 41598_2021_99656_MOESM3_ESM.pdf]

Supplementary Information

Efficient marmoset genome engineering by autologous embryo transfer and CRISPR/Cas9 technology

Yukiko Abe[1], Harumi Nakao[1], Motoki Goto[1], Moe Tamano[1], Michinori Koebis[1], Kazuki Nakao[1,2], Atsu Aiba[1].

[1]Section of Animal Research and Laboratory of Animal Resources, Center for Disease Biology and Integrative Medicine, Graduate School of Medicine, The University of Tokyo, Tokyo, Japan.

[2]Institute of Experimental Animal Sciences, Graduate School of Medicine, Osaka University, Osaka, Japan.

Correspondence

K. Nakao, Institute of Experimental Animal Sciences, Graduate School of Medicine, Osaka University, 2-2 Yamadaoka, Suita, Osaka 565-0871, Japan.

Tel: +81-6-6879-3100

E-mail: k_nakao@iexas.med.osaka-u.ac.jp

A. Aiba, Laboratory of Animal Resources, Center for Disease Biology and Integrative Medicine, Graduate School of Medicine, The University of Tokyo, 7-3-1 Hongo, Bunkyo-ku, Tokyo 113-0033, Japan.

Tel: +81-3-5841-3638

E-mail: aiba@m.u-tokyo.ac.jp

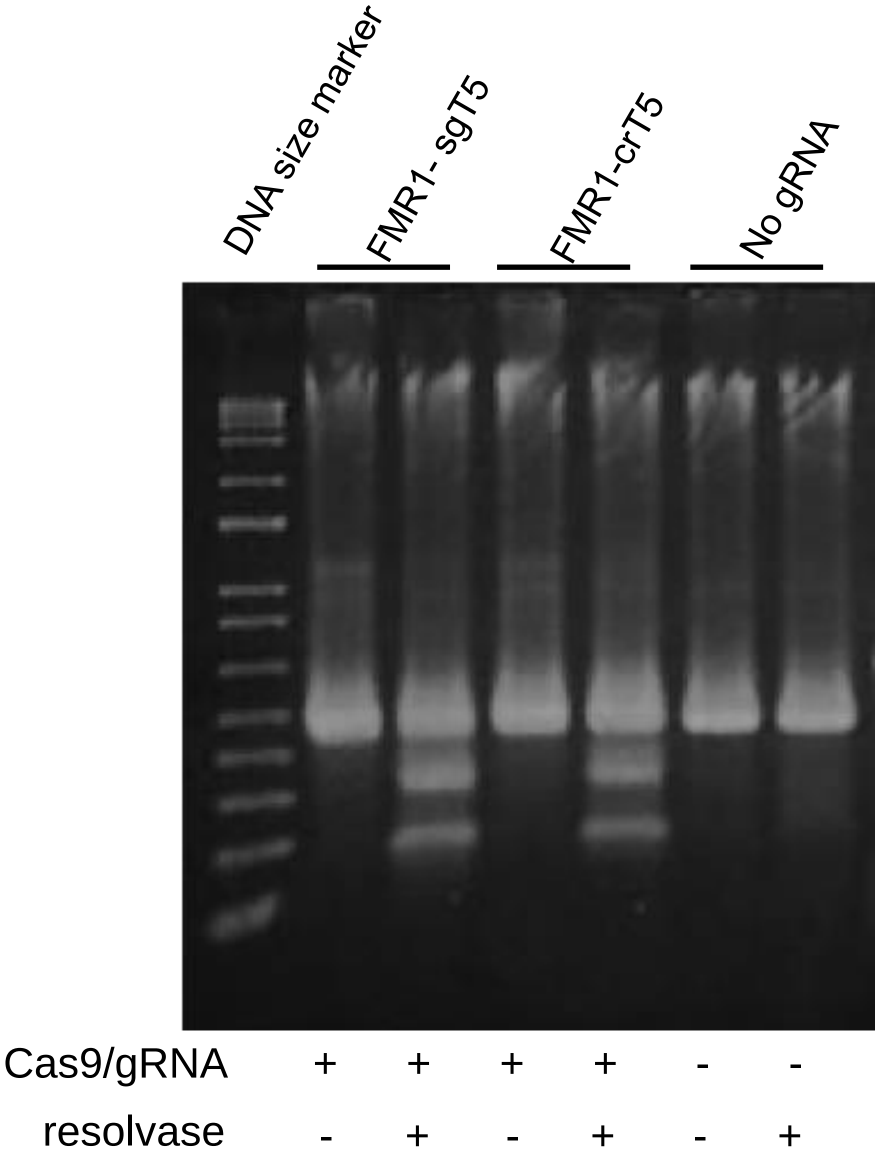

Supplementary Figure S1. Validation of gRNA for *FMR1* gene
Genomic DNA was extracted from marmoset ES cells transfected with SpCas9 protein/gRNA complexes. The PCR-amplified products containing a target site were denatured and annealed. When a mismatch is generated by annealing, the annealed DNA is cleaved by resolvase. Both sgRNA (FMR1-sgT5) and crRNA/tracrRNA (FMR1-crT5) showed significant amount of cleaved DNAs, suggesting that Cas9 with either gRNAs efficiently introduced mutations in the target site of the *FMR1* gene. The two lanes (No gRNA) on the far right show the PCR products from cells transfected with Cas9 protein only.

.

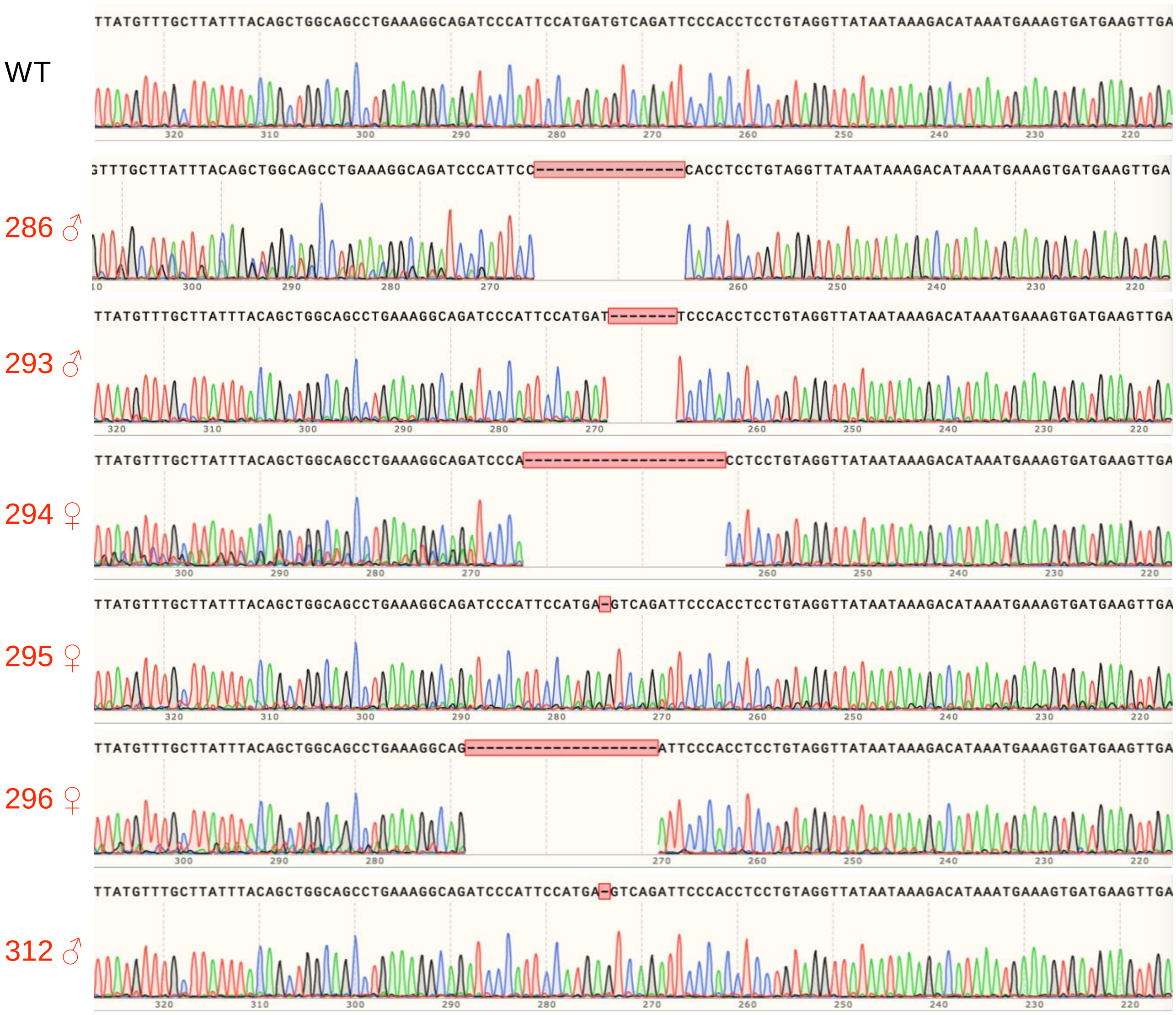

Supplementary Figure S2. DNA sequences of *FMR1* mutations in newborns

The genomic sequences of the *FMR1* target sites of wild-type (WT) and mutants are shown. Deletions of 15 bp, 7 bp, 21 bp, 1 bp, 20 bp and 1 bp were observed in #286, #293, #294, #295, #296 and #312, respectively. A male mutant #286 showed at least two waveforms, suggesting the mosaicism. The WT data is from #292, a sibling of #293.

a

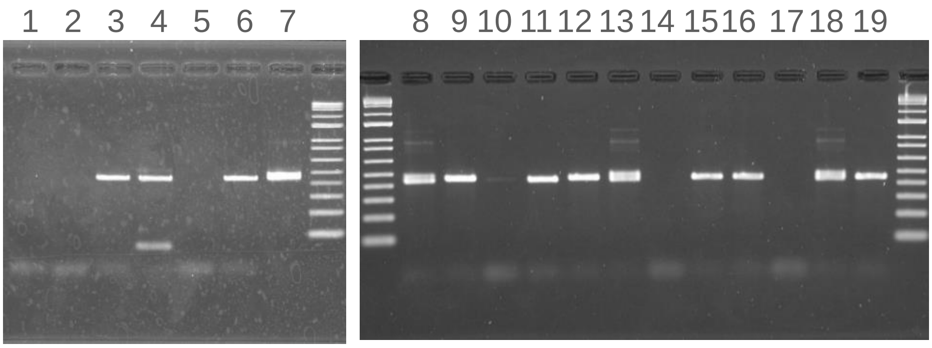

b

286-11

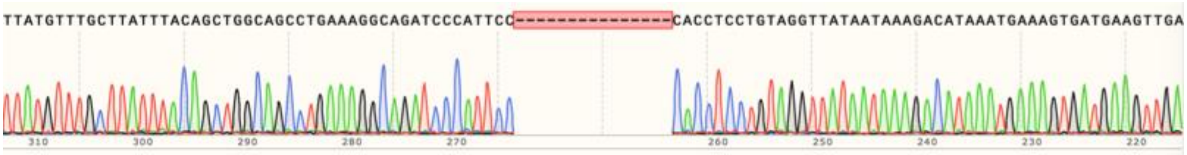

Supplementary Figure S3. PCR and DNA sequencing analysis of spermatozoa of #286 mosaic male

(a) Agarose gel electrophoreses of PCR products from spermatozoa from #286 male marmoset. Each lane contains a PCR product from a single spermatozoon.

(b) DNA sequence analysis of lane 11 sample. 15 bp deletion observed in #286 hair follicle genome was confirmed in the genome of the spermatozoon, suggesting a germ-line transmission of the *FMR1* mutant allele.

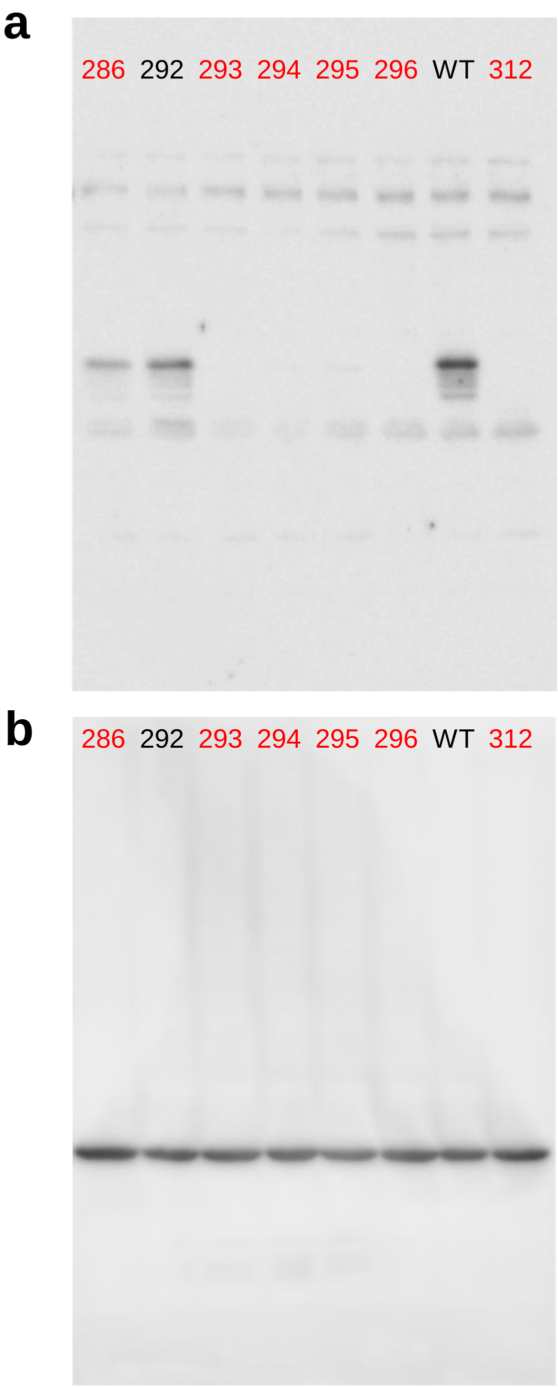

Supplementary Figure S4. Western blot analysis of FMRP and β-actin

Proteins extracted from fibroblasts of newborns (#286, #292, #293, #294, #295, #296, and #312) were separated on 8% polyacrylamide gels. Red numbers indicate mutants. All lanes contain 10 µg of protein. A membrane was reacted with the FMRP antibody (a) and then reprobed with β-actin antibody (b).

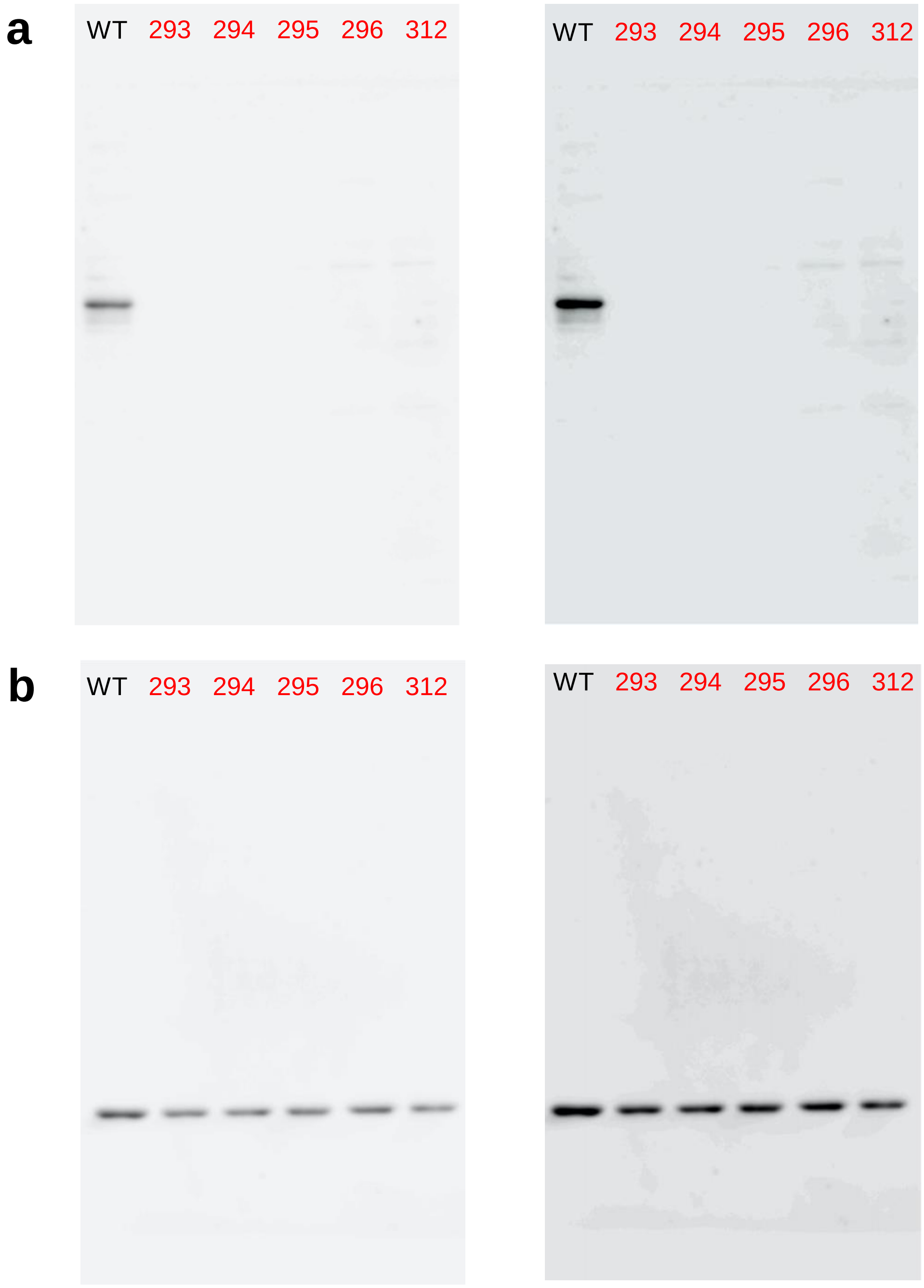

Supplementary Figure S5. Western blot analysis of FMRP and β-actin

Proteins extracted from whole brains of mutant newborns (#293, #294, #295, #296, and #312) were separated on 8% polyacrylamide gels. All lanes contain 10 µg of protein. A membrane was reacted with the FMRP antibody (a) and then reprobed with β-actin antibody (b).

Left: shorter exposure time, Right: longer exposure time.

NO.		day8	day9	day10	day11	day12	day13
1*	P4	1.64			2.2	7.8	
	E2	184.4			<L	20	
	sperm	+	—	—	+	+	
2	P4	1.68			2.8		
	E2	100			20		
	sperm	+	—	—	+		
3	P4	1			3.44		
	E2	487.6			191.6		
	sperm	+	+	+	+		
4	P4	2.56			4.04	8.6	
	E2	<L			202.8	20	
	sperm	—	—	—	—	+	
5	P4	1.68			3.8		
	E2	189.6			20		
	sperm	+	+	+	+		
6	P4	1.72			2.16		
	E2	141.6			20		
	sperm	+	+	+	+		
7	P4	2.04			6.86		
	E2	181			333.4		
	sperm	+	+	+	+		
8	P4	0.64			1.6	2.04	
	E2	<L			159.6	102	
	sperm	+	+	+	+	+	
9	P4	2.36			3.36		
	E2	162.8			20		
	sperm	+	+	+	+		
10	P4	2.88			5		
	E2	<L			100.8		
	sperm	+	+	+	+		
11	P4	1.76			2	3.4	
	E2	<L			424.8	20	
	sperm	+	—	—	+	+	
12*	P4	1.52			1.64	4.2	
	E2	<L			152	20	
	sperm	+	—	+	+	+	
13	P4	1.26			1.88	2.4	5.24
	E2	638.8			196.4	<L	20
	sperm	+	—	—	—	+	+
14	P4	1.04	2.08	1.68	6.4		
	E2	89.6	262.8	656.4	104.8		
	sperm	+	+	+	+		
15*	P4	9.24	5.24	10.2	19.6		
	E2	734.8	120.8	<L	20		
	sperm	—	—	+	+		
16	P4	4.2	1.4	1.64	3.2	3.52	
	E2	170.4	167.6	203.2	256.4	20	
	sperm	+	+	+	+	+	
17	P4	1.08	0.92	2.6	3		
	E2	288	374.4	916.4	133.2		
	sperm	+	+	+	+		
18	P4	2.84	2.28	1.72	2.4	1.8	8.84
	E2	120	133.6	175.2	261.2	640.8	482
	sperm	—	+	+	+	+	+

Supplementary Table S1. [P4] (ng/ml) and [E2] (pg/ml) and sperm in the vagina of females used for AET.

AET was performed on the day shown in red for each marmoset. <L indicates below detection limit. *Females which did not provide pronuclear stage embryos.
