# Supplementary figures and images for: Efficient marmoset genome engineering by autologous embryo transfer and CRISPR/Cas9 technology
Source: Sci Rep. 2021 Oct 12;11:20234. doi: 10.1038/s41598-021-99656-4 (PMC8511084; doi:10.1038/s41598-021-99656-4)

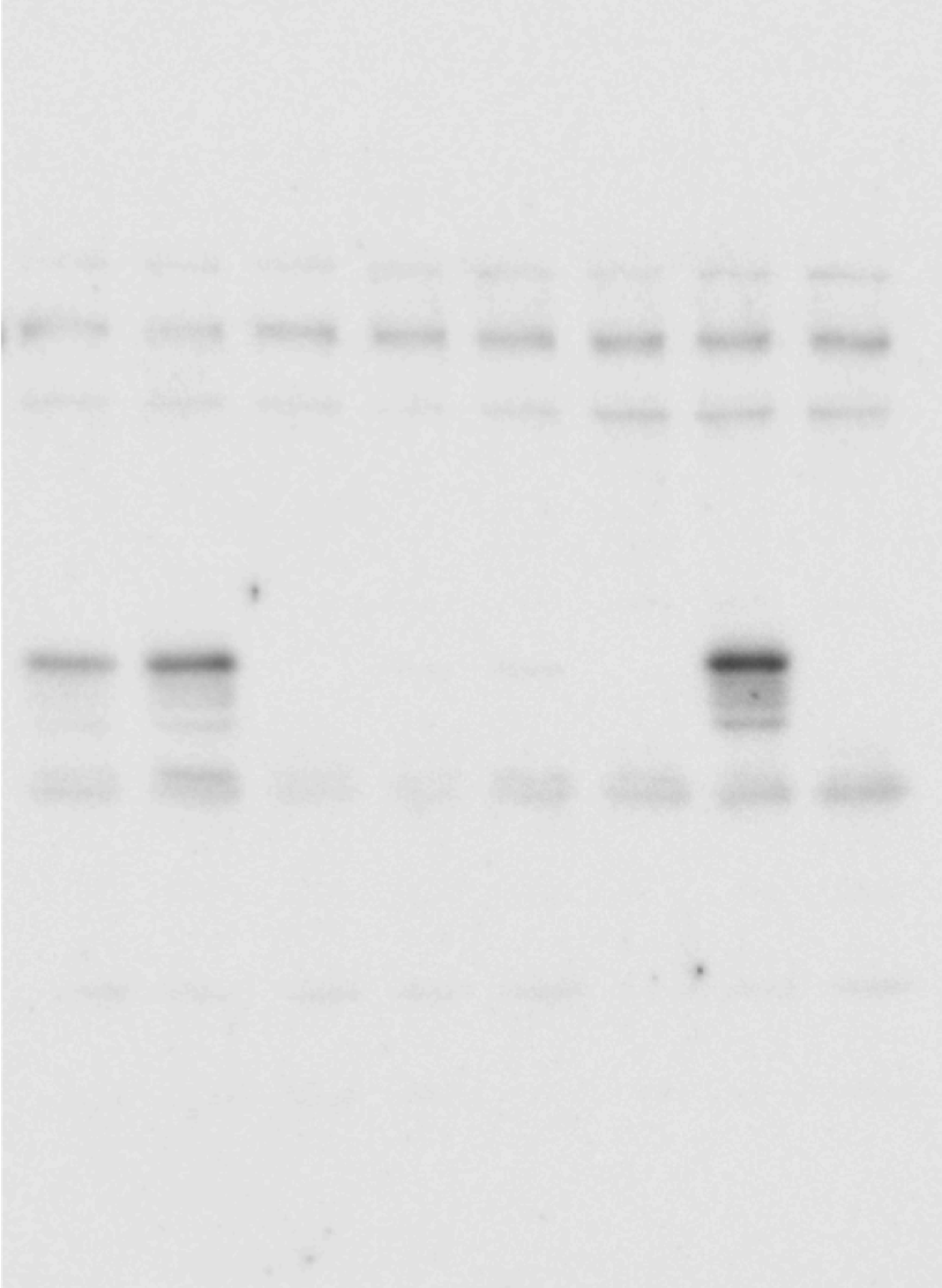

Supplement: Supplementary file 4 — Supplementary Information 2. [file 41598_2021_99656_MOESM4_ESM.png]

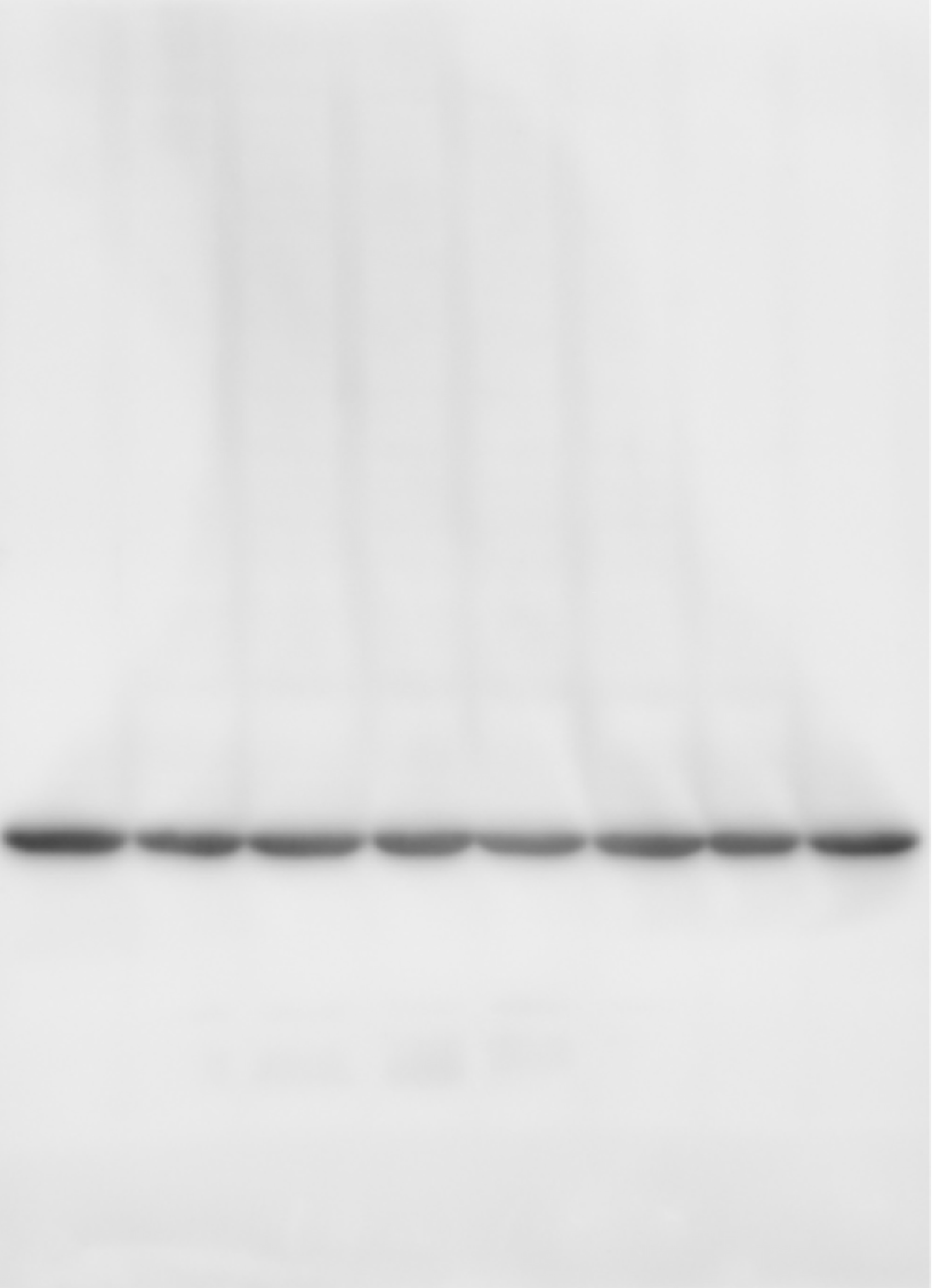

Supplement: Supplementary file 5 — Supplementary Information 3. [file 41598_2021_99656_MOESM5_ESM.png]

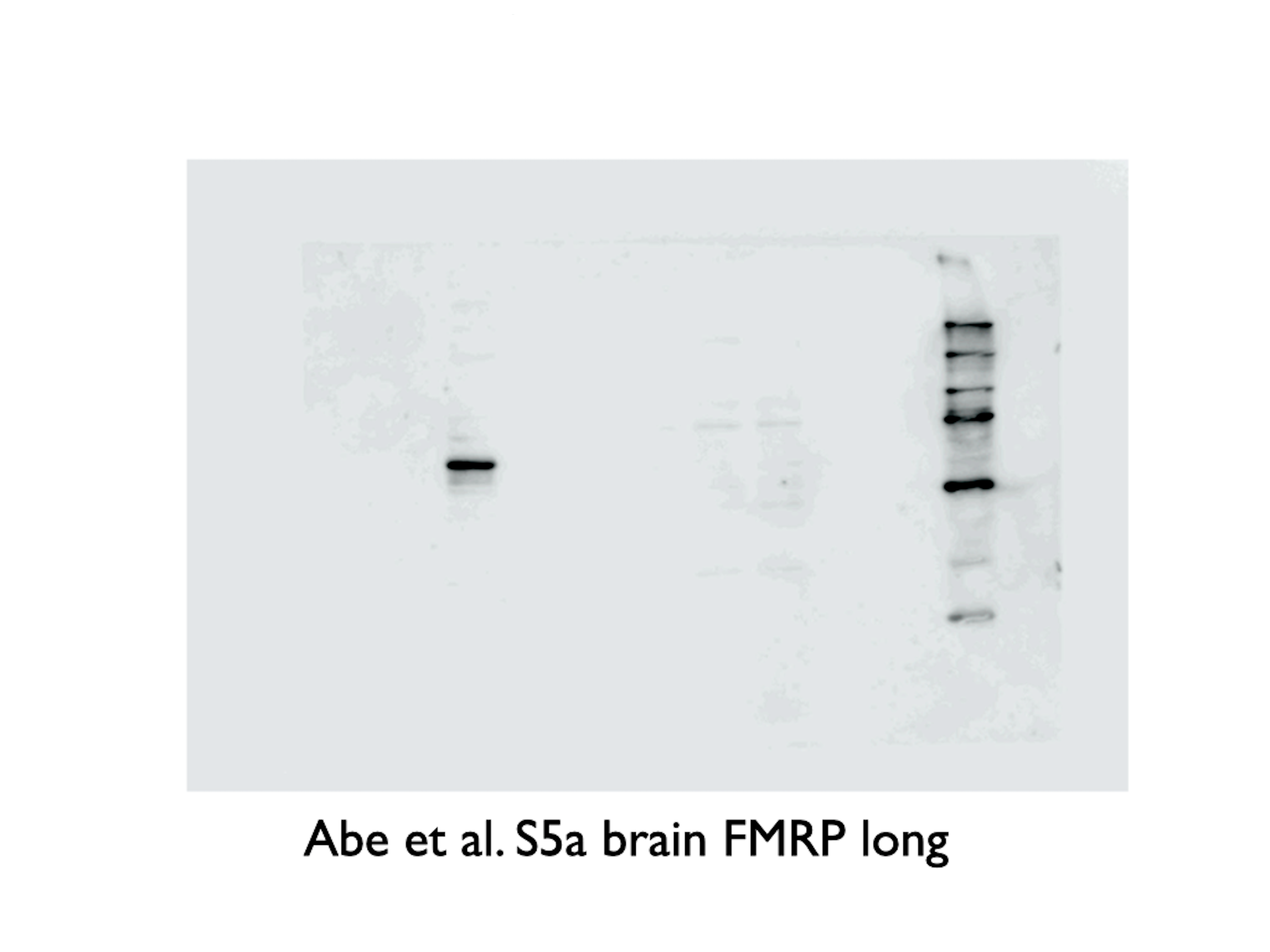

Supplement: Supplementary file 6 — Supplementary Information 4. [file 41598_2021_99656_MOESM6_ESM.tiff]

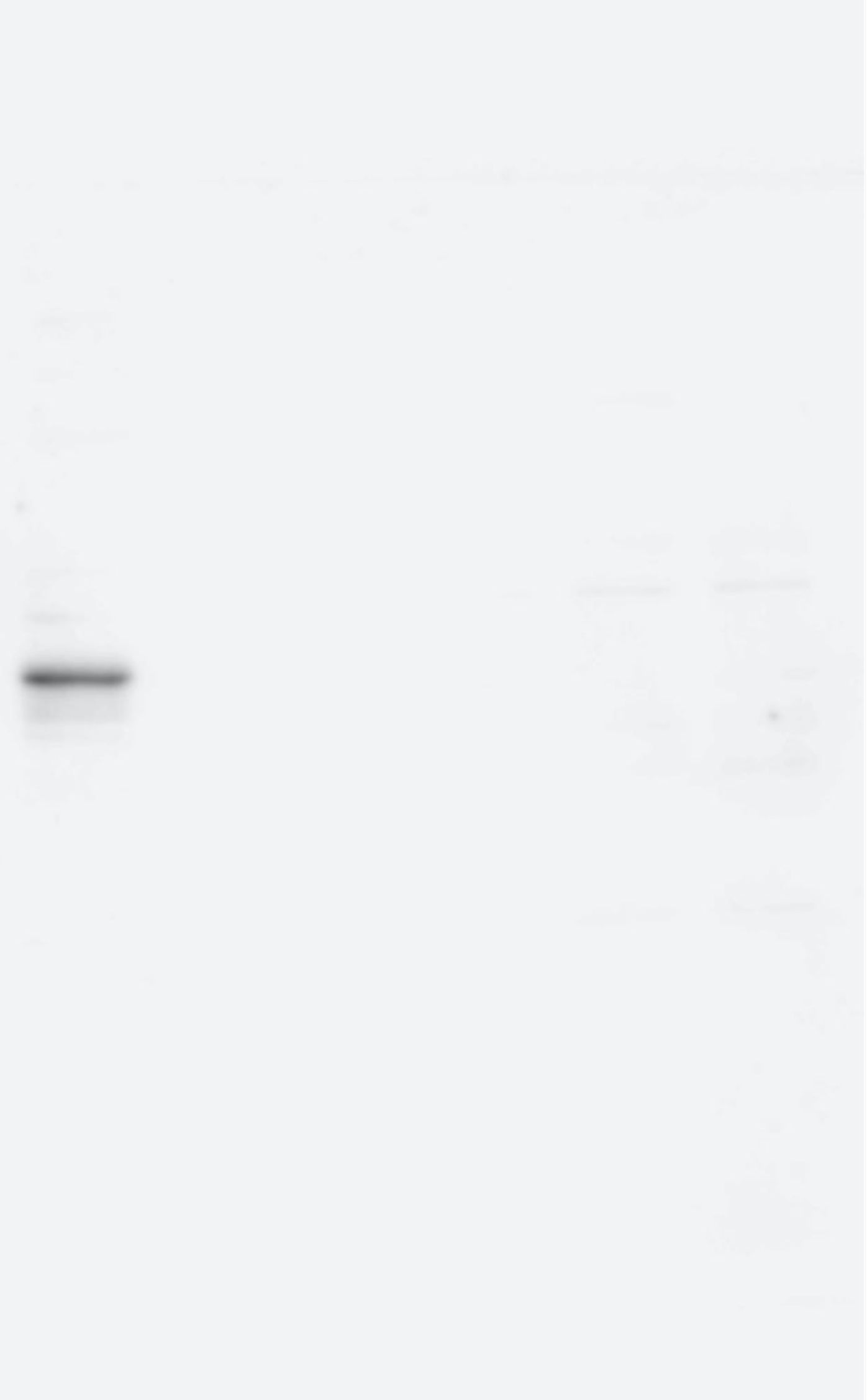

Supplement: Supplementary file 7 — Supplementary Information 5. [file 41598_2021_99656_MOESM7_ESM.png]

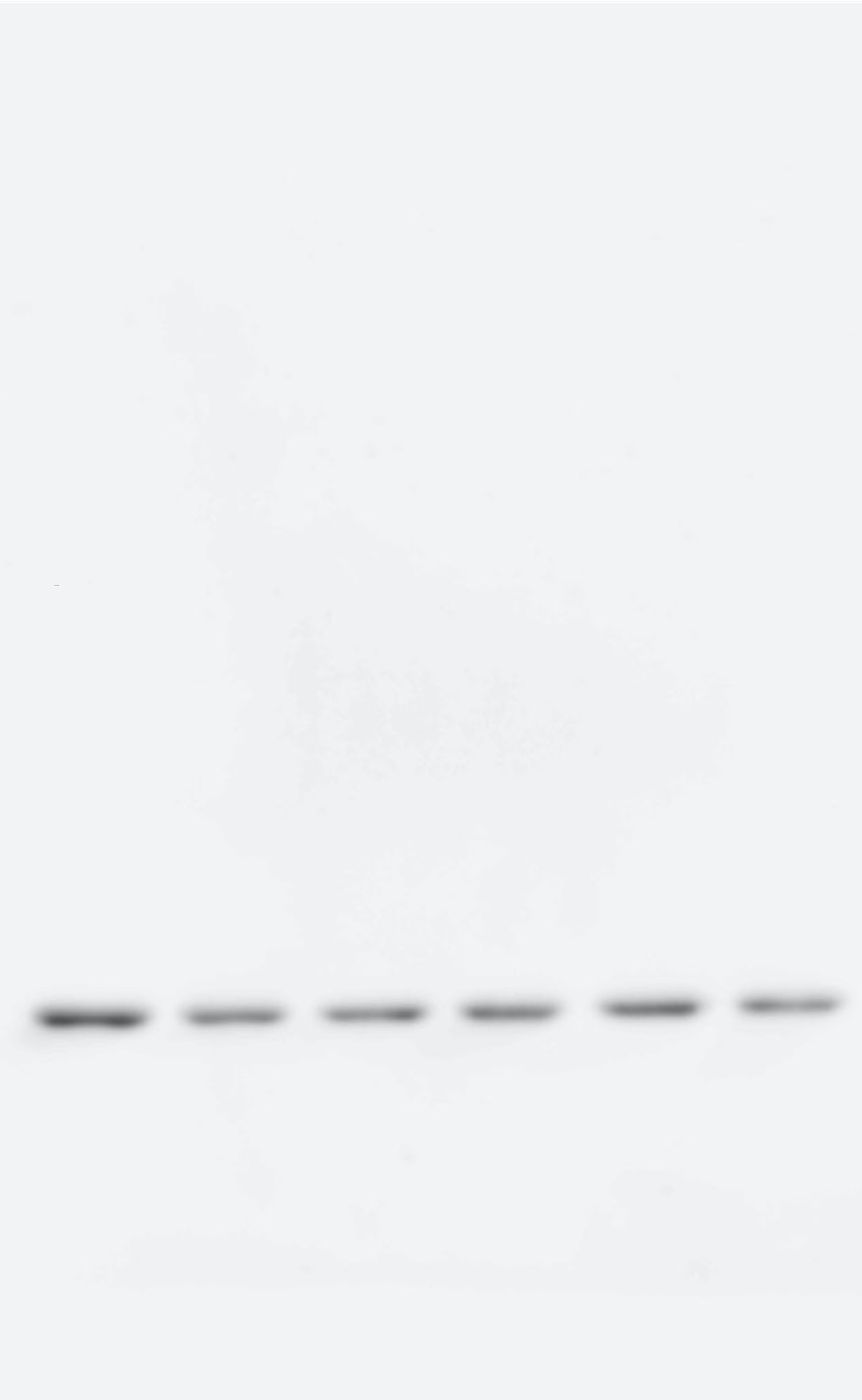

Supplement: Supplementary file 8 — Supplementary Information 6. [file 41598_2021_99656_MOESM8_ESM.png]

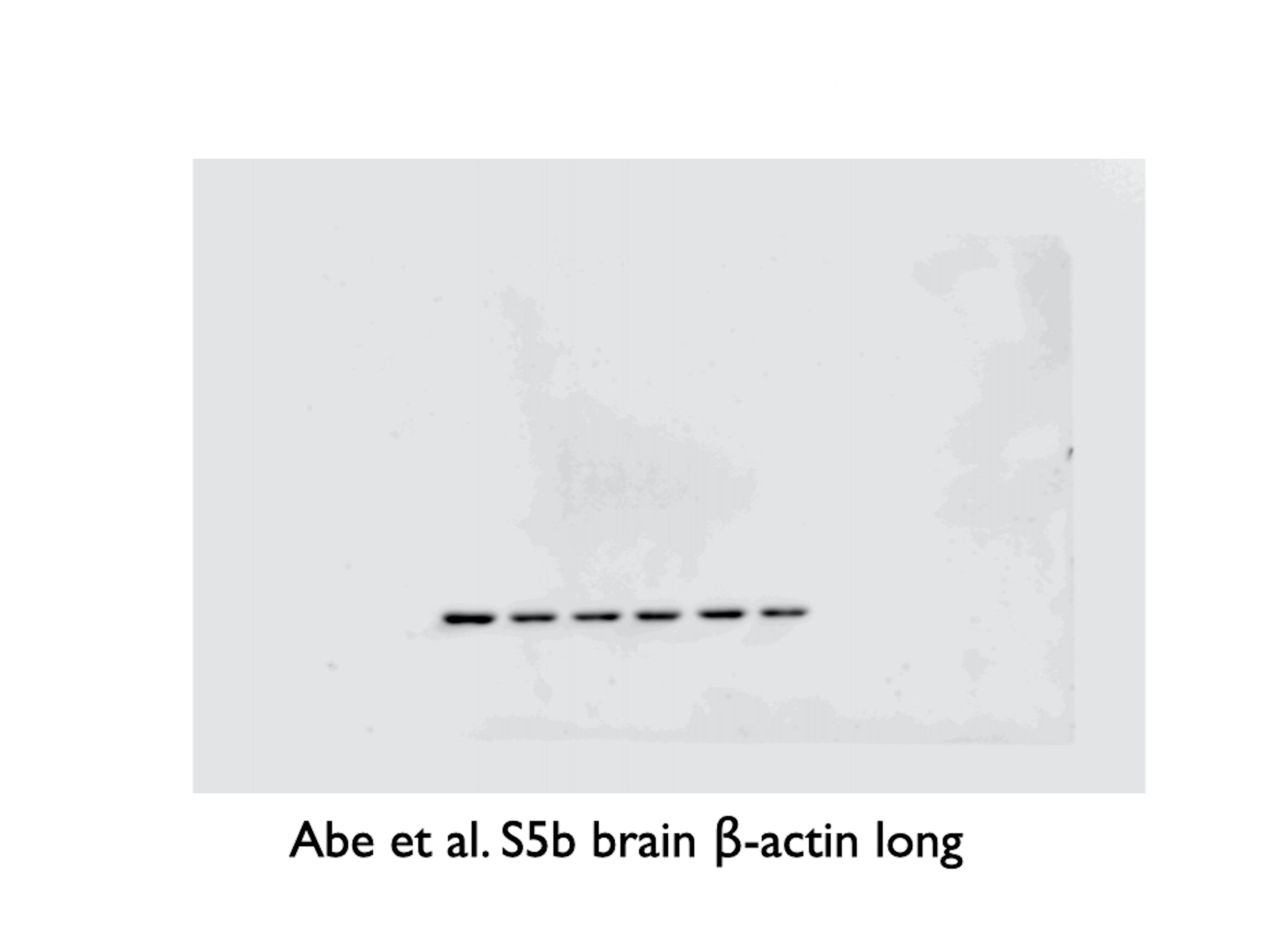

Supplement: Supplementary file 9 — Supplementary Information 7. [file 41598_2021_99656_MOESM9_ESM.tiff]
